# Comparison of adenocarcinoma and adenosquamous carcinoma prognoses in Chinese patients with FIGO stage IB-IIA cervical cancer following radical surgery

**DOI:** 10.1186/s12885-020-07148-x

**Published:** 2020-07-16

**Authors:** Xiaojing Zhang, Zunfu Lv, Xiaoxian Xu, Zhuomin Yin, Hanmei Lou

**Affiliations:** 1 Department of Gynecological Oncology, Cancer Hospital of University of Chinese Academy of Sciences (Zhejiang Cancer Hospital), Institute ofCancer Research and Basic Medicine (IBMC), Chinese Academy of Sciences, 1 Banshan East Road, Hangzhou, 310022 P. R. China; 2 Department of Agriculture and Food Science, Zhejiang A&F University, Lin’an, 311300 P. R. China

**Keywords:** Adenocarcinoma, Adenosquamous carcinoma, Survival, Radiotherapy, Concurrent chemoradiotherapy

## Abstract

**Background:**

To compare adenocarcinoma (AC) and adenosquamous carcinoma (ASC) prognoses in patients with FIGO stage IB–IIA cervical cancer who underwent radical hysterectomy.

**Methods:**

We performed a retrospective analysis of 240 patients with AC and 130 patients with ASC. Kaplan–Meier curves, Cox regression models, and log-rank tests were used for statistical analysis.

**Results:**

Patients with ASC had higher frequencies of lymphovascular space invasion (LVSI) and serum squamous cell carcinoma antigen (SCC-Ag) > 5 ng/ml (*p* = 0.049 and *p* = 0.013, respectively); moreover, they were much older (*P* = 0.029) than patients with AC. There were no clinically significant differences in overall survival (OS) between the groups. When stratified into three risk groups based on clinicopathological features, survival outcomes did not differ between patients with AC and those with ASC in any risk group. Multivariate analysis showed that lymph node metastasis (LNM) was an independent risk factor for recurrence-free survival (RFS) and OS in patients with AC and in patients with ASC. Carcinoembryonic antigen (CEA) > 5 ng/ml and SCC-Ag > 5 ng/ml were independent predictors of RFS and OS in patients with AC. In addition, among those stratified as intermediate-risk, patients with ASC who received concurrent chemoradiotherapy (CCRT) had significantly better RFS and OS (*P* = 0.036 and *P* = 0.047, respectively).

**Conclusions:**

We did not find evidence to suggest that AC and ASC subtypes of cervical cancer were associated with different survival outcomes. CCRT is beneficial for survival in intermediate-risk patients with ASC, but not in those with AC. Serum tumour markers can assist in evaluating prognosis and in providing additional information for patient-tailored therapy for cervical AC.

## Background

There were an estimated 570,000 cases of cervical cancer, including 311,000 deaths, worldwide in 2018. Cervical cancer is the fourth most frequently diagnosed cancer and the fourth leading cause of cancer death in women [1]. If cervical cancer is caught in the early stages [International Federation of Gynecology and Obstetrics (FIGO) stage I–II], the 5-year survival rate is generally at least 80% [2].

For patients with FIGO stage IB–IIA cervical cancer, radical radiation therapy or radical hysterectomy plus pelvic lymphadenectomy (RH-PLND) are the primary treatments. Primary radical surgery for most early stage cervical cancers is preferred, particularly for adenocarcinoma (AC) [3]. This is because it allows for more accurate surgical staging and avoids chronic radiation injury. After surgical resection, adjuvant radiotherapy (RT) or concurrent chemoradiotherapy (CCRT) is recommended depending on the patient-specific pathologic risk factors [4]. In cervical cancer, the most common sites of distant metastasis are the lung, bone, and liver.

Cervical cancer comprises three common histologic subtypes: squamous carcinoma (SCC), AC, and adenosquamous carcinoma (ASC). While the most common histologic type of cervical cancer is SCC, which constitutes approximately 75% of all cases, it is progressively decreasing in incidence [5]. Approximately 20–25% of cervical carcinomas are AC, the second most common histologic type [6, 7]; its incidence is increasing, particularly in women aged 20–40 years [8]. Due to the relative rarity of AC and ASC, optimal management and prognostic factors for early-stage patients have not been clearly established. Currently, ACs and ASCs (AC/ASCs) are treated similarly to SCC [9, 10].

Controversy exists regarding whether histologic type can have an impact on the prognosis of cervical cancer. Previous studies identified similar outcomes among patients with AC, ASC, or SCC [11–18]. However, some studies have shown that ASC histology is associated with a worse prognosis, compared to that of AC histology [19, 20], other studies found that early-stage AC and ASC are more aggressive and have worse prognoses, compared to SCC [9, 21–24]. Given that only a few studies have directly compared outcomes between patients with AC and those with ASC [11, 20], the relationship between histology findings (AC or ASC) and the outcome of cervical cancer remains unclear. We therefore evaluated outcomes and prognostic factors in patients with FIGO stage IB-IIA AC or ASC, after radical hysterectomy followed by tailored adjuvant therapy.

## Methods

### Study population

We examined the records of Chinese patients with stage IB-IIA AC or ASC, who received primary radical treatment and RH-PLND at Zhejiang Cancer Hospital from January 2010 to December 2016.No patients received neoadjuvant chemotherapy or RT prior to surgery. There were 435 patients with complete clinical data and 65 patients were excluded due to a lack of follow-up information.

### Pathologic characteristics and adjuvant therapy

Clinicopathologic data were collected, including tumour size, histotype, grade of differentiation, lymph node metastasis (LNM), depth of cervical stromal invasion (DSI), lymphovascular space invasion (LVSI), parametrial invasion (PI), resection margin status, and distant metastasis. Recurrence-free survival (RFS) was calculated as the number of months from the date of surgery to either the date of recurrence or the date of censoring. Overall survival (OS) was calculated as the number of months from the date of surgery to either the date of death or the date of censoring. Preoperative serum levels of squamous cell carcinoma antigen (SCC-Ag), CA125, CEA, and CA19–9 were detected using an automatic chemiluminescence immunoassay analyser. Cut-off levels for cancer antigens recommended by detection kit manufacturers were 1.5 ng/ml for SCC-Ag, 5 ng/ml for carcinoembryonic antigen (CEA), 37 U/ml for carbohydrate antigen (CA)19–9, and 35 U/ml for CA 125. The clinical cut-off value applied for SCC-Ag in this study was 5 ng/ml, defined by maximising the log-rank statistics for OS in the total population.

High-risk patients were defined as those with pathological findings, including LNM, PI, and positive results in the margin of the vagina. LVSI, DSI, and a tumour size ≥4 cm were the criteria for intermediate-risk status. CCRT was generally administered to such high-risk patients, while the low-risk group were observed only. Intermediate-risk patients generally underwent CCRT or conventional external beam radiotherapy (EBRT) of the pelvis (1.8–2.0 Gy for 25–27 days. No patient received brachytherapy. The RT regimen was the same for CCRT. The chemotherapy regimen consisted of weekly cisplatin (40 mg/m^2^) for 4–5 cycles, or paclitaxel (135 mg/m^2^) with cisplatin (60 mg/m^2^) every 3 weeks for 1–2 cycles.

### Statistical analysis

To identify prognostic factors for RFS and OS, the correlation between clinicopathologic factors and RFS or OS were analysed and compared between the AC and ASC groups. Survival rates and differential survival were estimated using Kaplan–Meier curves and log-rank tests. Univariate Cox regression and stepwise multivariate Cox regression using the forward Wald method were performed to determine independent prognostic factors for survival. The proportional hazards assumption was tested based on the Schoenfeld residual. All *P* values in this study were two-sided, and *P*-values < 0.05 were considered statistically significant. All data were analysed using SPSS statistical software (version 22.0; IBM Corp., Armonk, NY, USA).

## Results

A total of 370 patients met the eligibility criteria for this study, including 240 (64.9%) with AC and 130 (35.1%) with ASC. The maximum follow-up period was 110 months. The treatment regimen for patients included cancer-directed surgery alone and cancer-directed surgery with adjuvant treatment (RT or CCRT). The mean follow-up period was 81 months (range: 8–110 months) for the AC group and 79 months (range: 13–96 months), for the ASC group. The 3- and 5-year OS rates for all patients were 78.2 and 70.5%, respectively, compared to 76.4 and 68.1%, respectively, for patients with AC, and 80.6 and 74.7%, respectively, for those with ASC.

### Characteristics and clinicopathological features of the patients

**Fig. 1:**
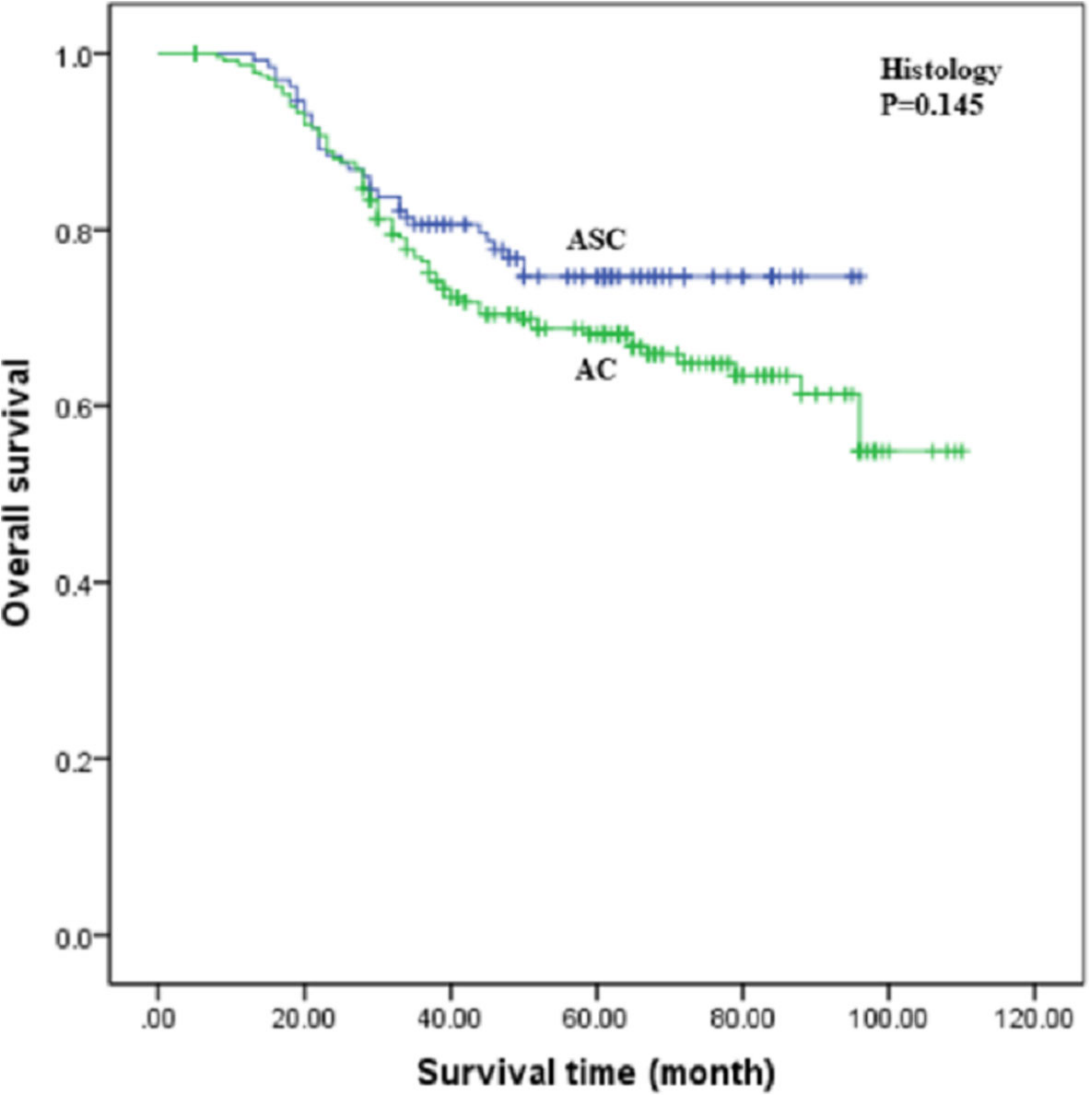
Kaplan-Meier curves of overall survival for patients with adenocarcinoma (AC) and adenosquamous carcinoma (ASC)

The clinicopathological features of the 370 eligible patients are summarised in Table 1. Cox regression analyses revealed that FIGO stage, tumour size, DSI ≥ 1/2, LNM, SCC-Ag, and CEA were significantly associated with OS. When the patients were stratified by histology with AC and ASC, no statistically significant differences were found between the groups in terms of OS (*P* = 0.145, Fig. 1). After adjustment for factors that were significant in univariate analysis, multivariate analysis showed that FIGO stage (HR = 1.83, 95% CI = 1.12–2.95) and LNM were significantly associated with shorter OS (HR = 2.29, 95% CI = 1.90–4.32). Clinicopathological features and OS were compared between patients with AC and those with ASC. As shown in Table 2, LVSI (*P* = 0.049) and SCC-Ag > 5 ng/ml (*P* = 0.013) were more common in the ASC group than in the AC group. Patients with ASC were older (> 40 years) than patients with AC (83.1% vs. 72.5%, *P* = 0.029). The differences in OS between patients with otherwise similar clinicopathological features were not statistically significant.

**Table 1:** Clinicopathological features associated with overall survival

Characteristics		No.	Overall survival	P		P
			Univariate analysis		Multivariate analysis	
			HR (95% CI)		HR (95% CI)	
Age	≤40	88	1			
	>40	282	1.33 (0.84–2.11)	0.220		
FIGO	IB	262	1		1	
	IIA	108	2.11 (1.45–3.07)	<0.001	1.83 (1.12–2.95)	0.003
Size	<4 cm	247	1		1	
	≥4 cm	123	1.70 (1.17–2.48)	0.006	1.56 (0.94–2.09)	0.083
LNM	No	267	1		1	
	Yes	103	3.43 (2.36–4.99)	<0.001	2.29 (1.90–4.32)	<0.001
LVSI	No	200	1			
	Yes	170	1.32 (0.91–1.92)	0.142		
DSI	<1/2	195	1		1	
	≥1/2	175	2.23 (1.51–3.28)	<0.001	2.16 (0.73–2.78)	0.079
SCC-Ag	≤5 ng/ml	351	1		1	
	>5 ng/ml	19	2.12 (1.07–4.21)	0.031	1.44 (0.71–3.56)	0.113
CEA	≤5 ng/ml	285	1		1	
	>5 ng/ml	85	1.83 (1.22–2.74)	0.004	1.13 (0.66–1.51)	0.076
CA 19–9	≤37 U/ml	311	1			
	>37 U/ml	59	1.38 (0.86–2.22)	0.183		
CA 125	≤35 U/ml	283	1			
	>35 U/ml	87	1.71 (0.98–3.00)	0.061		
RT	No	165				
	Yes	205	0.80 (0.55–1.71)	0.251		
Histology	AC	240				
	ASC	130	0.725 (0.478–1.10)	0.145

**Table 2:** Clinicopathologic features in the AC and ASC groups

Characteristics		AC	ASC	P^a^	Overall survival	
		(*n* = 240)	(*n* = 130)		HR (95% CI)	P^b^
Age	≤40	66	22	0.029	0.67 (0.23–1.98)	0.471
	>40	174	108		0.70 (0.45–1.11)	0.129
Size	<4 cm	162	85	0.729	0.77 (0.44–1.33)	0.345
	≥4 cm	78	45		0.65 (0.34–1.24)	0.192
LNM	No	172	95	0.809	0.64 (0.35–1.18)	0.153
	Yes	68	35		0.88 (0.50–1.56)	0.660
LVSI	No	139	61	0.049	0.71 (0.38–1.33)	0.289
	Yes	101	69		0.71 (0.40–1.24)	0.223
DSI	<1/2	135	60	0.065	0.57 (0.26–1.24)	0.158
	≥1/2	105	70		0.71 (0.43–1.18)	0.187
SCC-Ag	≤5 ng/ml	233	118	0.013	0.72 (0.46–1.11)	0.138
	>5 ng/ml	7	12		0.34 (0.09–1.28)	0.110

P^a^-value, clinicopathological features were compared between patients with AC and those with ASC; HR and P^b^-value, OS were compared between patients with AC and those with ASC using log-rank tests

### Survival analysis of patients with AC and ASC

**Fig. 2:**
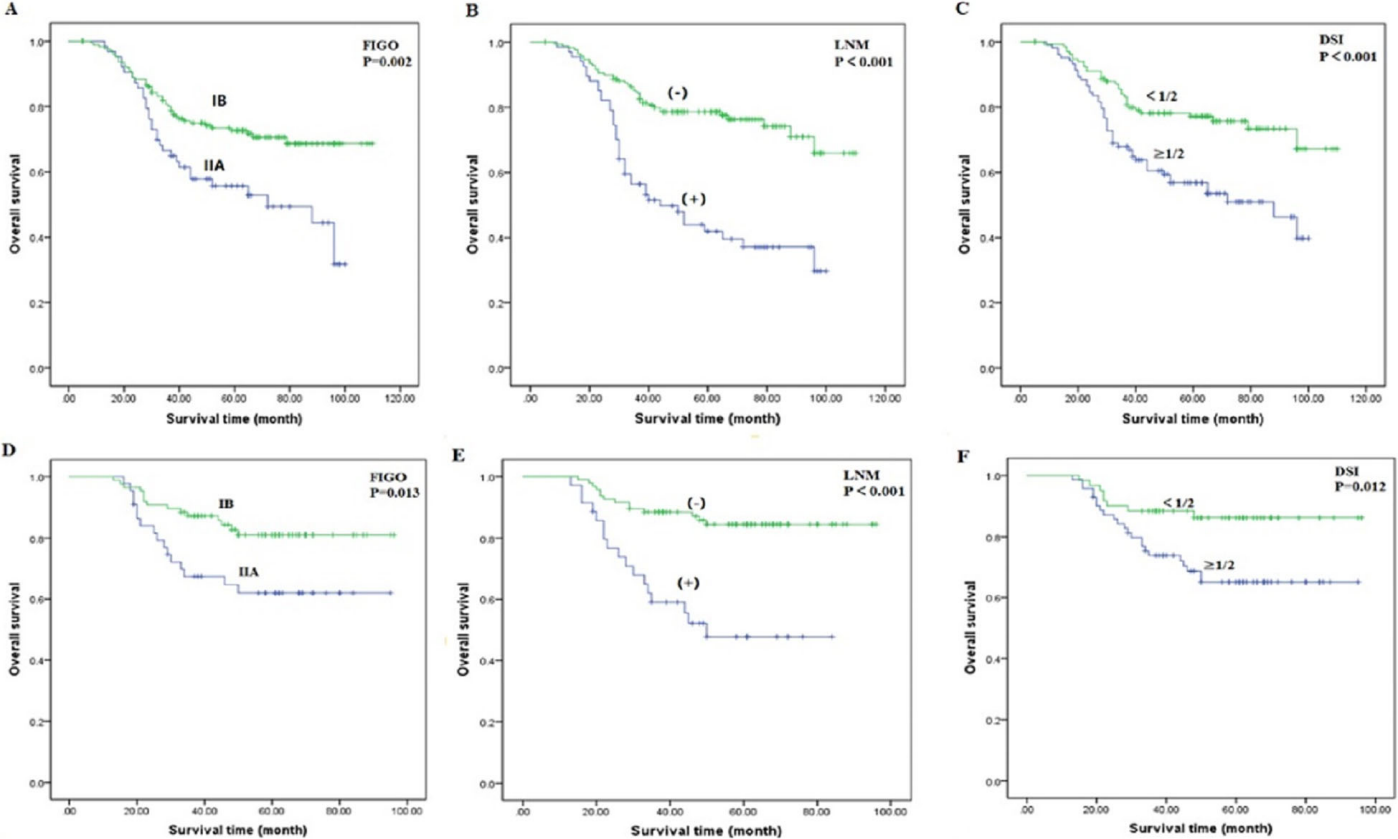
Kaplan-Meier curves of overall survival for patients with adenocarcinoma (**a**, **b** and **c**) and adenosquamous carcinoma (**d**, **e** and **f**) by FIGO stage, LNM and DSI

**Fig. 3:**
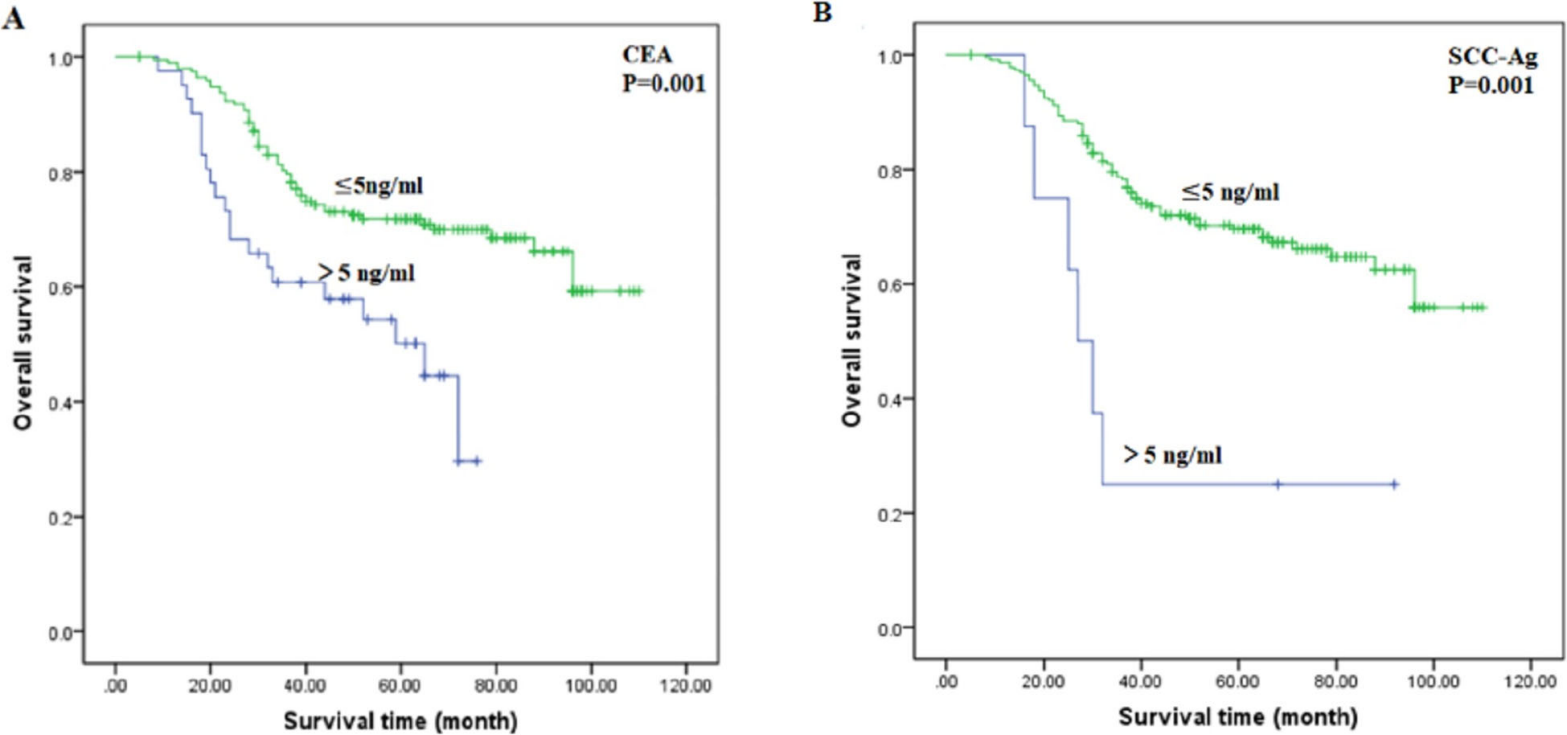
Kaplan-Meier curves of overall survival for patients with adenocarcinoma by CEA and SCC-Ag

As shown in Tables 3 and 4, univariate Cox regression analyses revealed that, as FIGO stage and DSI increased, and lymph node metastasis, whereas RFS and OS significantly decreased, among patients with ASC and AC (Fig. 2). In the AC group, RFS and OS were significantly associated with tumour size (*P* = 0.011 and *P* = 0.06, respectively), CEA (*P* = 0.023 and *P* = 0.001, respectively; Fig. 3), SCC-Ag (*P* = 0.012 and *P* = 0.001, respectively; Fig. 3), and CA 125 (*P* = 0.036 and *P* = 0.060, respectively); the associations were not significant in the ASC group. CA 125 (*P* = 0.0036) was associated with OS in patients with AC, but not with RFS (*P* = 0.060).

**Table 3:** Clinicopathological features associated with survival outcomes of AC

Characteristics		No.	RFS	P	OS	P
			5-year rate (%)		5-year rate (%)	
Age	≤40	66	69.9	0.262	74.0	0.229
	>40	174	63.4		66.1	
FIGO Stage	IB	176	78.1	0.003	72.7	0.002
	IIA	64	59.7		56.0	
Size	<4 cm	162	71.3	0.011	73.2	0.006
	≥4 cm	78	55.2		57.7	
LNM	No	172	70.5	<0.001	73.0	<0.001
	Yes	68	40.4		41.9	
LVSI	No	139	69.9	0.149	72.4	0.159
	Yes	101	60.2		62.8	
DSI	<1/2	135	75.9	<0.001	79.9	<0.001
	≥1/2	105	52.4		53.5	
SCC-Ag	≤5 ng/ml	233	67.7	0.012	69.7	0.001
	>5 ng/ml	7	25.0		25.0	
CEA	≤5 ng/ml	199	70.2	0.023	71.8	0.001
	>5 ng/ml	41	47.6		50.1	
CA 19–9	≤37 U/ml	198	67.7	0.248	70.1	0.195
	>37 U/ml	42	58.6		59.3	
CA 125	≤35 U/ml	185	70.3	0.060	71.5	0.036
	>35 U/ml	55	53.8		54.2

**Table 4:** Clinicopathological features associated with survival outcomes of ASC

Characteristics		No.	RFS	P	OS	P
			5-year rate (%)		5-year rate (%)	
Age	≤40	22	78.0	0.374	80.0	0.459
	>40	108	71.2		73.6	
FIGO Stage	IB	86	77.6	0.035	81.0	0.013
	IIA	44	58.2		62.0	
Size	<4 cm	85	75.5	0.413	77.5	0.282
	≥4 cm	45	66.8		69.6	
LNM	No	95	81.7	<0.001	84.4	<0.001
	Yes	35	42.4		47.8	
LVSI	No	61	76.9	0.156	77.6	0.453
	Yes	69	71.3		72.0	
DSI	<1/2	60	81.3	0.039	83.2	0.012
	≥1/2	70	63.7		65.1	
SCC-Ag	≤5 ng/ml	118	72.8	0.595	75.8	0.291
	>5 ng/ml	12	62.6		64.2	
CEA	≤5 ng/ml	86	75.9	0.441	77.4	0.205
	>5 ng/ml	44	67.1		69.0	
CA 19–9	≤37 U/ml	113	71.6	0.536	74.5	0.886
	>37 U/ml	17	72.5		75.3	
CA 125	≤35 U/ml	98	72.4	0.256	78.5	0.070
	>35 U/ml	32	60.7		62.2

Multivariate Cox regression analysis revealed that the combination of CEA > 5 ng/ml (*P* = 0.042 and *P* = 0.033 for RFS and OS, respectively), SCC-Ag > 5 ng/ml (*P* = 0.027 and *P* = 0.018 for RFS and OS, respectively), and LNM (*P* = 0.001, and *P* = 0.001 for RFS and OS, respectively) was a significant predictor of poor survival in patients with AC. Only LNM (*P* = 0.026 and *P* = 0.001 for RFS and OS, respectively) was a significant predictor of poor survival in patients with ASC.

The 5-year RFS and OS rates in the low-, intermediate-, and high-risk groups were 77.2 and 80.8%; 75.1 and 77.4%; and 35.1 and 41.8%, respectively, for the AC group, and 83.7 and 85.4%, 80.5 and 83.7%, and 39.9 and 47.8% respectively, for the ASC group (Table 5). There was no statistically significant difference in RFS or OS between patients with AC and those with ASC in any risk group (*P* > 0.05, Table 6).

**Table 5:** Stratified analysis of risk group associated with survival outcomes of AC and ASC

	No.	AC	5-year (%)	OS	P
		RFS	P		
High risk group	68	38.5	<0.001	41.9	<0.001
Intermediate risk group	112	74.2		77.4	
Low risk group	60	79.1		80.8	
		ASC	5-year (%)		
	No.	RFS	P	OS	P
High risk group	35	44.6	<0.001	47.8	<0.001
Intermediate risk group	65	81.2		83.7	
Low risk group	30	83.9		85.4

**Table 6:** Survival analysis by histologic type and risk group

	No.	RFS	P	OS	P
		5-year (%)		5-year (%)	
High risk group |			0.563		0.675
AC	15	38.5		41.9	
ASC	4	44.6		47.8	
Intermediate risk group					
AC	14	74.2	0.549	77.4	0.647
ASC	9	81.2		83.7	
Low Risk Group					
AC	89	79.1	0.485	80.8	0.437
ASC	28	83.9		85.4	

### Effect of adjuvant therapy on intermediate-risk-group patients

According to the univariate analysis, intermediate-risk ASC patients who received CCRT had significantly better RFS and OS than those who received no further treatment (NFT) (HR = 0.101, 95% CI = 0.011–0.939; and HR = 0.108, 95% CI = 0.012–0.972 for RFS and OS, respectively). Although the hazard ratio for RFS with RT alone was statistically significant (HR = 0.691, 95%CI = 0.192–0.981), that was not true of OS (HR = 0.760, 95% CI = 0.204–1.434). Patients with AC who received RT or CCRT did not have better RFS or OS than those who received NFT (Table 7).

**Table 7:** Stratified analysis of treatment associated with survival of AC and ASC

		AC					ASC			
		RFS		OS			RFS		OS	
Treatment	No.	HR(95% CI)	P	HR(95% CI)	P	No.	HR(95% CI)	P	HR(95% CI)	P
NFT	53	1	0.745	1	0.951	15	1	0.043	1	0.134
RT	21	1.068 (0.314–3.017)		1.095 (0.387–3.104)		22	0.691 (0.192–0.981)		0.760 (0.204–1.434)	
CRRT	38	0.764 (0.334–2.063)		0.920 (0.393–2.154)		28	0.101 (0.011–0.939)		0.108 (0.012–0.972)	

## Discussion

The main histological type of cervical cancer is SCC [25]. However, the incidences of AC and ASC of the uterine cervix have increased over the past 40 years, especially among younger women [26–29]. In this retrospective cohort study, we examined the records of Chinese patients with FIGO stage IB-IIA AC or ASC to evaluate potential prognostic factors among these patients. All patients underwent surgery as the primary treatment. Multivariate analyses showed that FIGO stage and LNM were independent prognostic factors for OS.

Previous studies showed that FIGO stage, tumour size, and LNM were independent prognostic factors for survival [13, 30, 31]. Shu et al. [2] reported that in patients with AC/ASC, differentiation was an independent predictor of OS, and LVSI was an independent predictor of DFS. We investigated whether histology (AC vs. ASC) is a prognostic factor in patients with cervical cancer. There were no differences, in terms of clinical impact on OS, between the two histological groups in early-stage cervical cancer, although a greater proportion of patients with ASC had LVSI and SCC-Ag > 5 ng/ml; moreover, patients with ASC were much older than those with AC. Multivariate Cox regression analysis revealed that CEA > 5 ng/ml and SCC-Ag > 5 ng/ml were independent risk factors for RFS and OS in patients with AC, but not in patients with ASC. This suggests that pre-treatment levels of CEA > 5 ng/ml and SCC-Ag > 5 ng/ml can be regarded as risk factors for AC, providing additional information for patient-tailored therapy, and should be analysed in prospective studies. Previous studies reported that elevated pre-treatment serum SCC-Ag levels were associated with poor prognosis [32, 33], but the histologic type of most patients was cervical squamous cell carcinoma a few patients had AC. Nakamura et al. [34] showed that AC, DOI, tumour size, and LVSI were significantly associated with disease recurrence.

The respective 5-year survival rates for patients with stages IB and IIA were 72.7 and 56% for the AC group and 81.0 and 62% for the ASC group. Saigo et al. [35] reported that 5-year survival rates for patients with stages I and II (IIA, IIB) AC were 79 and 37%, respectively. Presumably because the latter group also included patients with IIB cancer, the 5-year survival rate for patients with stage II cancer was lower than the rate observed in our study. These results suggest that as FIGO stage increases, the survival time is reduced accordingly. Our results demonstrated that FIGO stage (IB vs. IIA) was significantly associated with survival time (*P* < 0.05). Similarly, Noh et al. [30] reported that ASC histology was associated with more favourable survival outcomes, compared to AC histology, although the differences were not statistically significant. Lai et al. [12] found no differences between the ASC and AC subtypes in RFS or cancer-specific survival (CSS).

Wang et al. [36] demonstrated that higher tumour grade and more vascular invasion were present in patients with ASC, compared to patients with AC. Reis et al. [11] found that Grade III histology and LVSI were more common in patients with ASC than in patients with AC. In addition, they demonstrated that although the time to recurrence was shorter for patients with ASC (7.9 months vs. 15 months; *P* = 0.01), differences in OS or recurrence rates between patients with AC and patients with ASC were not statistically significant. Baek et al. [18] reported greater mean tumour size and more frequent LVSI in patients with ASC, but found that histologic type did not influence RFS or OS in multivariate analyses, following adjustment for significant prognostic factors. In contrast, several studies reported poor survival for patients with ASC. A meta-analysis by Lee et al. [16] demonstrated that ASC patients may have poorer outcomes than those with AC of the cervix. Farley et al. [20] observed an increased risk of death among patients with ASC histology compared to those with AC histology. Twu et al. [37] found that survival for ASC was slightly worse than that for AC in a univariate analysis, but the RFS and CSS of the two subtypes were not significantly different in multivariate analyses.

Our study demonstrated a tendency for better RFS and OS in patients with ASC than in those with AC, in both the low-risk and intermediate/high-risk groups, although the difference was not statistically significant. The prognosis for the ASC subtype appears to be intermediate (i.e. between those of the SCC and AC subtypes) [31]. Previous studies showed no statistically significant differences between patients with AC and those with ASC in low-, intermediate-, or high-risk groups (*P* > 0.05) [9, 18]. However, Lea et al. reported that ASC histology was associated with reduced disease-free survival relative to AC histology, among patients with low-risk stage IB1 cancer [38].

We also examined the effect of treatment on OS in intermediate-risk ASC and AC patients. Univariate analysis indicated that in patients with ASC, CCRT was associated with significantly better RFS and OS. RT alone was related to RFS but not OS. This indicated that RT alone may be effective for local control, while CCRT is advantageous for control of distant metastasis. In addition, RT and CCRT did not confer any survival benefit in patients with AC. This may have been because there is greater radio resistance and more aggressive behaviour of tumours in patients with AC relative to those with ASC. A retrospective study suggested that RT and CCRT after radical hysterectomy were not beneficial in intermediate-risk patients. In particular, RT and CCRT appeared to increase the incidence of lymphedema, and even led to RT-related morbidities such as small-bowel obstruction and leg oedema [34, 39]. Twu et al. confirmed that adjuvant therapy (radiotherapy with or without chemotherapy) following RH-PLND, for early stage AC/ASC patients with a low prognostic score, may not improve survival. Therefore, omitting adjuvant therapy could decrease morbidity [37]. We suspected that systemic CT alone could confer a survival benefit for patients with AC. Takekuma et al. [40] reported that chemotherapy alone after surgery for high-risk patients had similar efficacy to CCRT, but with less toxicity. Further prospective randomized studies including larger patient populations are needed to confirm our findings.

Our study was limited by its retrospective design. Furthermore, since most patients in the high-risk group received CCRT, while most patients in the low-risk group underwent observation only, the statistical power may not have been sufficient to detect a statistical difference in the impact of adjuvant therapies on survival. Finally, systemic CT alone, i.e., without RT, might confer a survival benefit. However, we did not investigate the effects of chemotherapy because no patient received systemic CT alone. Despite these limitations, to our knowledge this study included the largest number of FIGO stage IB–IIA cervical AC/ASC patients undergoing radical hysterectomy. It also provided sufficient data on prognosis and adjuvant treatment efficacy, given the long follow-up period.

## Conclusion

In conclusion, there were no differences, in terms of OS, between early stage AC and ASC cervical cancers. Patients with ASC were older (> 40 years) and more likely to have LVSI and SCC-Ag > 5 ng/ml, compared to patients with AC. LNM, CEA > 5 ng/ml, and SCC-Ag > 5 ng/ml were independent risk factors for poor RFS and OS in patients with AC, whereas only LNM was an independent risk factor for poor RFS and OS in patients with ASC. In addition, within an intermediate-risk-stratified group, patients with ASC who received CCRT experienced significantly better survival outcomes. Our findings may facilitate improvements in clinical diagnostics and therapeutic applications for patients with cervical cancer.

## Data Availability

The datasets used and analyzed during the current study are available from the corresponding author on reasonable request.

## Abbreviations

AC: adenocarcinoma
ASC: adenosquamous carcinoma
RFS: recurrence-free survival
OS: overall survival
LNM: lymph node metastasis
DSI: depth of stromal invasion
LVSI: lymph–vascular space invasion
MST: the median survival time
RT: radiotherapy
CCRT: concurrent chemoradiotherapy
CEA: Carcinoembryonic antigen
kSCC: squamous cell carcinoma antigen
CA: carbohydrate antigen (CA)
HR: hazard ratio

## References

[1] Bray F, Ferlay J, Soerjomataram I, Siegel RL, Torre LA, Jemal A, et-al. Global cancer statistics 2018: globocan estimates of incidence and mortality worldwide for 36 cancers in 185 countries. Ca A Cancer J Clin. 2018;68(6):394–424. 10.3322/caac.21492 30207593

[2] Shu T, Zhao D, Li B, Wang Y, Liu S, Li P (2017). Prognostic evaluation of postoperative adjuvant therapy for operable cervical cancer: 10 years’ experience of National Cancer Center in China. Chin J Cancer Res.

[3] Baalbergen A, Veenstra Y, Stalpers L (2013). Primary surgery versus primary radiotherapy with or without chemotherapy for early adenocarcinoma of the uterine cervix. Cochrane Database Syst.

[4] Ryu SY, Kim MH, Nam BH, Lee TS, Song ES, Park CY (2014). Intermediate-risk grouping of cervical cancer patients treated with radical hysterectomy: a korean gynecologic oncology group study. Br J Cancer.

[5] Fujiwara H, Yokota H, Monk B, Treilleux I, Devouassoux-Shisheboran M, Davis A (2014). Gynecologic cancer intergroup (GCIG) consensus review for cervical adenocarcinoma. Int J Gynecol Cancer.

[6] Young RH, Clement PB (2002). Endocervical adenocarcinoma and its variants: their morphology and differential diagnosis. Histopathology..

[7] Chan PG, Sung HY, Sawaya GF (2003). Changes in cervical cancer incidence after three decades of screening US women less than 30 years old. Obstet Gynecol.

[8] Vinh-Hung V, Bourgain C, Vlastos G, Gábor Cserni, Ridder, MD, Storme G, et al. Prognostic value of histopathology and trends in cervical cancer: a seer population study. BMC Cancer; 2007: 7(1), 164–0. 10.1186/1471-2407-7-164 PMC1994954 17718897

[9] Mabuchi S, Okazawa M, Matsuo K, Kawano M, Suzuki O, Miyatake T (2012). Impact of histological subtype on survival of patients with surgically-treated stage ia2–iib cervical cancer: adenocarcinoma versus squamous cell carcinoma. Gynecol Oncol.

[10] National Comprehensive Cancer Network NCCN Clinical Practice Guidelines in Oncology, Cervical Cancer Version 2 (2015).

[11] Reis RD, Frumovitz M, Milam MR, Capp E, Sun CC, Coleman RL (2007). Adenosquamous carcinoma versus adenocarcinoma in early-stage cervical cancer patients undergoing radical hysterectomy: an outcomes analysis. Gynecol Oncol.

[12] Lai CH, Chou HH, Chang CJ, Wang CC, Hsueh S, Huang YT (2013). Clinical implications of human papillomavirus genotype in cervical adeno-adenosquamous carcinoma. China J Modern Med.

[13] Kasamatsu T, Onda T, Sawada M, Kato T, Ikeda S, Sasajima Y (2009). Radical hysterectomy for FIGO stage I–IIB adenocarcinoma of the uterine cervix. Br J Cancer.

[14] Park JY, Kim DY, Kim JH, Kim YM, Kim YT, Nam JH (2010). Outcomes after radical hysterectomy in patients with early-stage adenocarcinoma of uterine cervix. Br J Cancer.

[15] Mabuchi S, Suzuki O, Kamiura S, Ogawa K, Kimura T, Okazawa M (2013). Impact of the addition of concurrent chemotherapy to pelvic radiotherapy in surgically treated stage IB1-IIB cervical cancer patients with intermediate-risk or high-risk factors: a 13-year experience. Int J Gynecol Cancer.

[16] Lee KBM, Lee JM, Park CY, Lee KB, Cho HY, Ha SY (2006). What is the difference between squamous cell carcinoma and adenocarcinoma of the cervix? A matched case–control study. Int J Gynecol Cancer.

[17] Chen JL, Cheng JC, Kuo SH, Chen CA, Lin MC, Huang CY (2012). Outcome analysis of cervical adenosquamous carcinoma compared with adenocarcinoma. Acta Obstet Gynecol Scand.

[18] Baek MH, Park JY, Kim D, Suh DS, Nam JH. Comparison of adenocarcinoma and adenosquamous carcinoma in patients with early-stage cervical cancer after radical surgery. Gynecologic Oncol. 2014;135(3):462–7. 10.1016/j.ygyno.2014.10.004 25312397

[19] Lee JY, Lee C, Hahn S, Kim MA, Kim HS, Chung HH (2014). Prognosis of Adenosquamous carcinoma compared with adenocarcinoma in uterine cervical Cancer: a systematic review and meta-analysis of observational studies. Int J Gynecol Cancer Official J Int Gynecol Cancer Soc.

[20] Farley JH, Hickey KW, Carlson JW, Rose GS, Kost ER, Harrison TA (2003). Adenosquamous histology predicts a poor outcome for patients with advanced-stage, but not early-stage, cervical carcinoma. Cancer.

[21] Nakanishi T, Ishikawa H, Suzuki Y, Inoue T, Nakamura S, Kuzuya K (2000). A comparison of prognoses of pathologic stage Ib adenocarcinoma and squamous cell carcinoma of the uterine cervix. Gynecol Oncol.

[22] Galic V, Herzog TJ, Lewin SN, Neugut AI, Burke WM, Lu YS (2012). Prognostic significance of adenocarcinoma histology in women with cervical cancer. Gynecol Oncol.

[23] Irie T, Kigawa J, Minagawa Y, Itamochi H, Sato S, Akeshima R (2000). Prognosis and clinicopathological characteristics of Ib-IIb adenocarcinoma of the uterine cervix in patients who have had radical hysterectomy. Eur J Surg Oncol.

[24] Huang YT, Wang CC, Tsai CS, Lai CH, Chang TC, Chou HH (2011). Long-term outcome and prognostic factors for adenocarcinoma/adenosquamous carcinoma of cervix after definitive radiotherapy. Int J Radiat Oncol Biol Phys.

[25] Gien LT, Beauchemin MC, Thomas G (2010). Adenocarcinoma: a unique cervical cancer. Gynecol Oncol.

[26] Sherman ME, Wang SS, Carreon J, Devesa SS (2005). Mortality trends for cervical squamous and adenocarcinoma in the United States. Cancer..

[27] Castellsagué X, Díaz M, De Sanjosé S, Muñoz N, Herrero R, Franceschi S (2006). Worldwide human papillomavirus etiology of cervical adenocarcinoma and its cofactors: implications for screening and prevention. J Natl Cancer Inst.

[28] Sasieni P, Adams J (2001). Changing rates of adenocarcinoma and adenosquamous carcinoma of the cervix in England. Lancet..

[29] Huang CY, You SL, Chen CJ, Cheng WF, Luo HC, Hsieh CY (2011). Incidence of cervical cancer and age-specific survival of small cell cervical carcinoma in Taiwan. Acta Obstet Gynecol Scand.

[30] Noh JM, Park W, Kim YS, Kim JY, Kim HJ, Kim J (2014). Comparison of clinical outcomes of adenocarcinoma and adenosquamous carcinoma in uterine cervical cancer patients receiving surgical resection followed by radiotherapy: a multicenter retrospective study (KROG 13-10). Gynecol Oncol.

[31] Chen JL‐Y, Cheng JC‐H, Kuo S‐H, Chen C‐A, Lin M‐C, Huang C‐Y. Outcome analysis of cervical adenosquamous carcinoma compared withadenocarcinoma[J]. Acta Obstetricia Et Gynecologica Scandinavica, 2012; 91(10):1158–1166. 10.1111/j.1600-0412.2012.01420.x 22497449

[32] Davelaar EM, Lande JVD, Mensdorff-Pouilly SV, Blankenstein MA, Kenemans PA (2008). Combination of serum tumor markers identifies high-risk patients with early-stage squamous cervical Cancer. Tumor Biol.

[33] Reesink-Peters N, van der Velden J, ten Hoor KA, Boezem HM, de Vries EGE, Schilthuis MS, Mourits MJE, Nijman HW, Aalders JG, Hollema H, Pras E, Duk JM, van der Zee AGJ (2005). Preoperative serum squamous cell carcinoma antigen levels in clinical decision making for patients with early-stage cervical cancer. J Clin Oncol.

[34] Nakamura K, Kitahara Y, Satoh T, Takei Y, Takano M, Nagao S (2016). Analysis of the effect of adjuvant radiotherapy on outcomes and complications after radical hysterectomy in FIGO stage IB1 cervical cancer patients with intermediate risk factors (GOTIC study). Gynecol Oncol.

[35] Saigo PE, Cain JM, Kim WS, Gaynor JJ, Johnson K, Jr LJ, et al. prognostic factors in adenocarcinoma of the uterine cervix. Cancer. 2004; 92(1):262–267. 10.1002/1097-0142(19860415)57:8<1584::aid-cncr2820570825>3.0.co;2-8 2418947

[36] Wang SS, Sherman ME, Silverberg SG, Carreon JD, Lacey JV, Zaino R (2006). Pathological characteristics of cervical adenocarcinoma in a multi-center US-based study. Gynecol Oncol.

[37] Twu NF, Ou YC, Liao CI, Chang WY, Yang LY, Tang YH (2016). Prognostic factors and adjuvant therapy on survival in early-stage cervical adenocarcinoma/adenosquamous carcinoma after primary radical surgery: a Taiwanese gynecologic oncology group (TGOG) study. Surg Oncol.

[38] Lea JS, Coleman RL, Garner EO, Duska LR, Miller DS, Schorge JO (2003). Adenosquamous histology predicts poor outcome in low-risk stage IB1 cervical adenocarcinoma. Gynecol Oncol.

[39] Lee KB, Lee JM, Ki KD, Lee SK, Park CY, Ha SY (2008). Comparison of adjuvant chemotherapy and radiation in patients with intermediate risk factors after radical surgery in FIGO stage IB-IIA cervical cancer. Int J Gynecol Cancer.

[40] Takekuma M, Kasamatsu Y, Kado N (2016). Adjuvant chemotherapy versus concurrent chemoradiotherapy for high-risk cervical cancer after radical hysterectomy and systematic lymphadenectomy. Int J Clin Oncol.

